# A New Porcelain for Prosthetic Purposes

**Published:** 1905-01

**Authors:** N. S. Jenkins

**Affiliations:** Dresden, Germany


### A NEW PORCELAIN FOR PROSTHETIC PURPOSES.[1]

BY DR. N. S. JENKINS, DRESDEN, GERMANY.

So soon as porcelain enamel had been proved to completely answer the requirements for restoring decayed or broken teeth to health, beauty, and usefulness, I began to occupy myself with the question of producing a porcelain which should simplify prosthesis and make porcelain crowns, bridges and continuous gum work easy to the general practitioner. It was a task, lightened, indeed, by previous experience, but presenting difficulties of no inconsiderable magnitude. The materials employed in making dental porcelain have hitherto been worked somewhat crudely. It has been rare to find a completed product sufficiently free from foreign matter or possessing the desired homogeneity. For this the manufacturers are not to blame. Their object has been to supply what they believed the profession to demand,—viz., cheap materials with which fairly good results can be obtained. No manufacturer, whose legitimate purpose is commercial, can be expected to carry on year-long experiments, at large expense, in the vague hope of supplying a superior material when he has no reason to suppose that there is, or will be, a demand for it. Our debt to the dental manufacturers is exceeding great, but there is much work which only a dentist in actual practice can do, for only he can know exactly what the practising dentist needs, and therefore it is vain and unreasonable to look to any other than a dentist to accomplish such work.

I have been so fortunately situated as to make experiments in porcelain my natural contribution to the progress of our pro-

---

[1] Read before The New York Institute of Stomatology, October 4, 1904.

fession, and it has been solely owing to these favorable circumstances that I am able to present to you to-night a product which seems to me to completely solve the question of porcelain prosthesis. For more than two years I have been able to produce it in the small quantities of laboratory experiment, but only recently has it been found possible to manufacture it in larger quantities. This delay is, however, no cause for regret. During this time I have not only been occupied with the question of manufacture, but also with the methods of applying this material, and those methods are now well systematized. At the very foundation lies the importance of a proper alloy of platinum-iridium. For plates, for caps and bands, ten per cent. of iridium is essential. For posts, twenty per cent. of iridium is necessary. Pure gold should be used for solder.

Prosthetic porcelain begins to melt at 950° C. The heat may be continued up to 1000° C., at which point the electric current should be suspended, and, usually, the piece can then be cooled rapidly. The melting-point of gold is, approximately, 1075° C. At a temperature of 1000° C. not only is there no danger of remelting the solder or volatilizing the gold, but under this heat the above alloy of platinum-iridium does not bend nor change its shape, and if this temperature is not exceeded, the life of the platinum wire of an electric furnace is greatly prolonged. The heat should be determined by a pyrometer. One cannot too greatly insist upon this point. Here is a simple, scientific method, through which it is possible to obtain exact results, without exception. No man who does this class of work, and every dentist ought to do it, can afford to leave anything to chance or to imagination. To be sure, through experience, one can easily learn to work prosthetic porcelain without a pyrometer, but, with the greatest care, the greatest expert will sometimes fail to recognize sudden changes in the electric current, which the pyrometer infallibly records.

Prosthetic porcelain may be used alone, but most dentists will find it desirable to use it in combination with artificial teeth. It combines perfectly with all porcelain teeth, in any proportion, from a delicate jacket crown to a massive molar. It can be used to supplement, to support, to extend, and to broaden teeth, or even to restore the lost glaze of an artificial tooth, with perfect confidence. It is far stronger than the strongest artificial teeth, and in a close bite can be safely used in a thickness of three millimeters. Crowns and bridges, which were formerly made of gold, can be made of platino-iridium, and so built out with prosthetic porcelain that no suggestion of artificiality can be discovered, and the work will be stronger and more cleanly. In fact, it enables any capable dentist to completely conceal his art in prosthesis and to restore the mouth of his patient to its original beauty and usefulness, for he can obtain, with certainty, any desired color, any necessary form, a density which makes grinding and subsequent polishing possible, enduring strength and hardness, and a brilliant and indestructible glaze. There is no limit to its usefulness or to the gratitude of the patients who have experienced its advantages, and it supplies one more step toward the proper remuneration of an overworked and underpaid profession.

