# Correction

**Published:** 1905-03

**Authors:** H. Carlton Smith


### Domestic Correspondence.

### CORRECTION.

To the Editor:

Sir,—In a recent issue of the International Dental Journal you printed a paper which I read last October before the Academy of Dental Science in Boston, the subject of which was, "Should the Dental Practitioner concern Himself with the Chemical Examination of Saliva or Urine." In this paper I gave some of the results of work with the micro-polariscope which has been done by Dr. Kirk, of your city, and Dr. Michaels, of Paris, but I find by some accident my preliminary references to these gentlemen were not published, making it perhaps appear that I claimed an originality in this line of investigation which did not belong to me. I have tried to be particular in this, and trust that you will correct any possible impression of this sort by an explanation in your journal.

My paper was written for the purpose of interesting dentists in Boston in a work which Dr. Kirk had previously interested me in, and which I am trying to carry on along lines laid down by Professor Michaels and Dr. Kirk, hence I do not want it to appear that such investigation was original with me when I was simply trying to extend an interest in the work of others, and at the same time, to be sure, to facilitate my own research.

H. Carlton Smith.

[The foregoing explanation is published with pleasure in justice to all parties interested, but the writer's assertion, "I find by some accident my preliminary references to these gentlemen" (Drs. Michaels and Kirk) "were not published," seems to carry the imputation that the editor of the International Dental Journal, or some one else, had presumed to eliminate an important part of the manuscripts. The author of the paper may rest assured that it *appeared in this journal without any change,* and the original copy received at this office gave no indication of having been subjected to the editing process elsewhere.—Editor.]

